# A Neurocomputational Approach to Trained and Transitive Relations in Equivalence Classes

**DOI:** 10.3389/fpsyg.2017.01848

**Published:** 2017-10-18

**Authors:** Ángel E. Tovar, Gert Westermann

**Affiliations:** ^1^Department of Psychology, Lancaster University, Lancaster, United Kingdom; ^2^Facultad de Psicología, Universidad Nacional Autónoma de México, Ciudad de Mexico, Mexico

**Keywords:** equivalence classes, transitive relations, neurocomputational model, Hebbian learning, categorization

## Abstract

A stimulus class can be composed of perceptually different but functionally equivalent stimuli. The relations between the stimuli that are grouped in a class can be learned or derived from other stimulus relations. If stimulus A is equivalent to B, and B is equivalent to C, then the equivalence between A and C can be derived without explicit training. In this work we propose, with a neurocomputational model, a basic learning mechanism for the formation of equivalence. We also describe how the relatedness between the members of an equivalence class is developed for both trained and derived stimulus relations. Three classic studies on stimulus equivalence are simulated covering typical and atypical populations as well as nodal distance effects. This model shows a mechanism by which certain stimulus associations are selectively strengthened even when they are not co-presented in the environment. This model links the field of equivalence classes to accounts of Hebbian learning and categorization, and points to the pertinence of modeling stimulus equivalence to explore the effect of variations in training protocols.

## Introduction

Humans (and some animals) can learn to group stimuli that share no physical similarity into arbitrary categories. For example, the utterance “Do not pass!,” a red light, and a road sign with a red rim and a person drawn in the center all indicate that I should not walk in a certain direction. The formation and processing of such arbitrary categories, also called *equivalence classes*, has been studied in detail in both humans and animals (Sidman et al., 1982; Sidman, 1994; Horne and Lowe, 1996; Zentall et al., 2002; O'Donnell and Saunders, 2003; Urcuioli and Swisher, 2015). This research has revealed two important characteristics of equivalence classes. First, it is not necessary to directly learn that all members in a category are equivalent to each other, but this knowledge can be derived from a number of directly learned relations. Second, the functional properties learned for one stimulus in a category can be transferred to the other stimuli without explicit training. For example, if a naïve human learns the meaning of “Do not pass!” and then learns the equivalence between this utterance and the red light, and the equivalence between the red light and the road sign, she will be able to derive the equivalence between the utterance and the road sign (*if* A equals B, and B equals C, *then* A equals C) which is called a transitive relation. She will also display the behavior learned for the utterance in the presence of the red light and the road sign. The question of how transitive relations are established between non-perceptually related stimuli has been long debated. In this paper, we show how transitive relations and equivalence classes are learned in an abstract neurocomputational model. The model provides a means to quantitatively approach the analysis of equivalence phenomena from the general principles of associative learning.

Equivalence classes have been systematically studied in behavioral psychology under the names of stimulus equivalence (SE), equivalence relations, or equivalence class formation. Most of the interest in SE is based on its relevance to analyze learned and derived stimulus-stimulus relations, which are fundamental for our understanding of symbolic behavior (Dugdale and Lowe, 1990; Sidman, 1994; Dickins and Dickins, 2001; Wilkinson and McIlvane, 2001). For example, the SE approach has been used to analyze correspondences between words, sounds and the objects they represent (Sidman, 1971); arbitrary visual symbols and meaningful stimuli (Nartey et al., 2015); and mathematical concepts and their graphical representations (Lynch and Cuvo, 1995; Fields et al., 2009).

A set of stimuli can be considered as equivalent if their relations exhibit the three definitional properties of mathematical equivalence: reflexivity, symmetry, and transitivity (Sidman and Tailby, 1982). These properties are commonly assessed via matching to sample tasks (MTS). In a regular MTS procedure, a participant learns to pick from several comparison stimuli (B1, B2… Bn) the one that correctly matches a sample stimulus (A1). For example, comparisons can be pictures of different animals and the sample can be the spoken name of a particular animal. The match between A1 and B1 (i.e., A1B1) can be learned if the selection of “B1” in the presence of the sample stimulus “A1” is followed by reinforcement.

If a participant has learned the matches A1B1 and B1C1 (nomenclature considers the first stimulus as the sample and the second as the correct comparison), equivalence is documented if, without any instruction or feedback, the participant selects A1 as comparison in the presence of A1 (reflexivity, A1*R*A1, with *R* denoting an equivalence relation); selects A1 in the presence of B1, and B1 in the presence of C1 (symmetry, e.g., *if* A*R*B, *then* B*R*A); and selects C1 in the presence of A1 (transitivity, *if* A*R*B, and B*R*C, *then* A*R*C). Symmetry and transitivity can be combined in what is called an *equivalence test* (Sidman, 1992): for the current example the equivalence test corresponds to selecting A1 as comparison when C1 is the sample.

**Figure 1:**
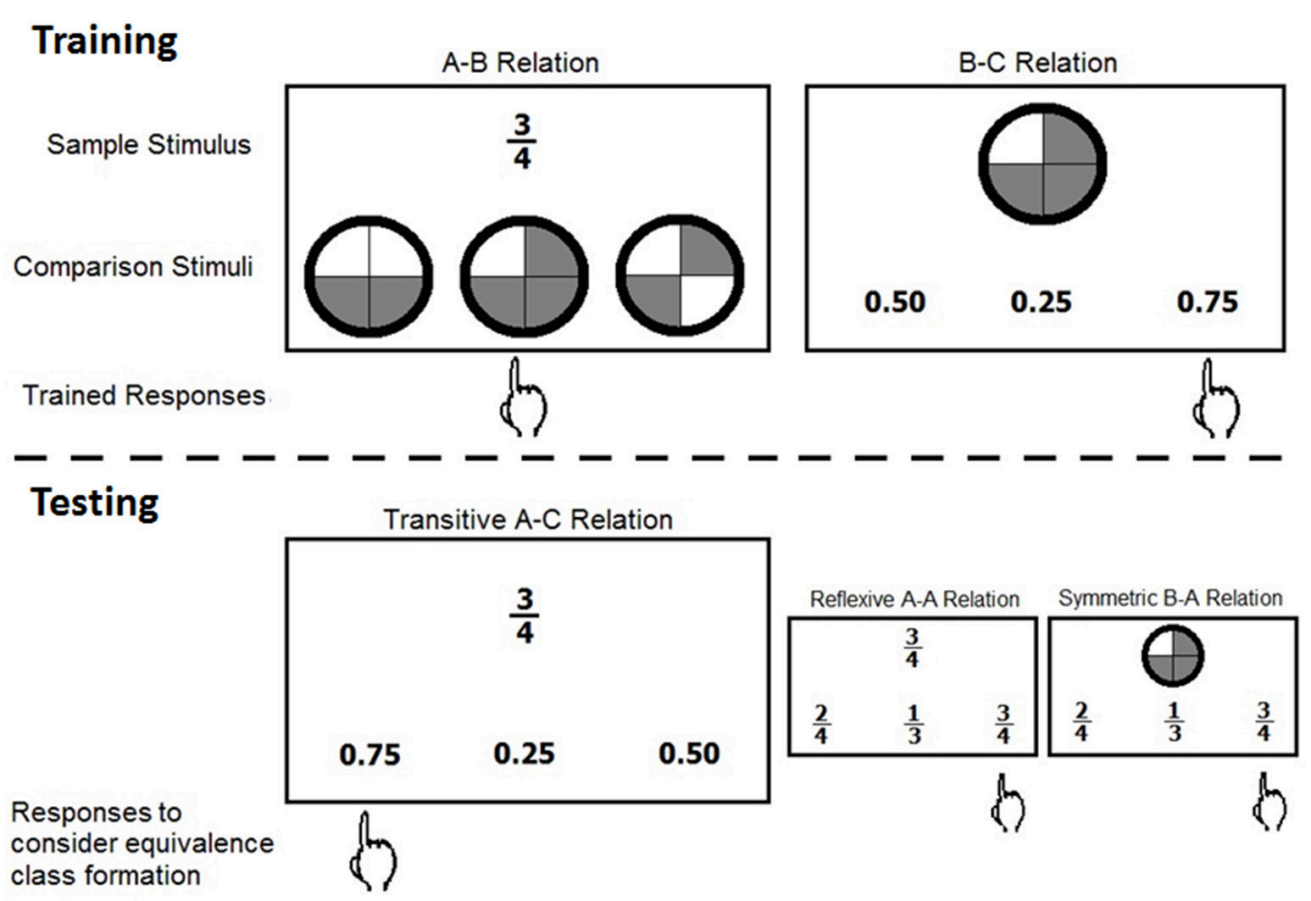
Examples of trained and derived stimulus relations in a matching to sample format. The sample stimulus, comparison stimuli, and trained and expected responses are shown for the training and testing phases. The stimuli A, B, and C correspond to different representations of the same quantity, only AB and BC are directly trained, AC, AA, and BA are examples of derived relations.

A typical SE study is divided into two phases, training and testing. During training, participants learn the *baseline relations* from which *emergent relations* can be derived and assessed during the testing phase. Figure 1 shows an example of trained and tested stimulus relations in a MTS format.

Transitive relations in SE are of particular interest: they challenge traditional approaches in behavior analysis to explain how two stimuli become functionally equivalent even when they have never been experienced together. Remarkably, the mechanisms underlying their establishment could inform our understanding of the properties of functional untrained behavior and the inclusion of members in categories that share no perceptual similarity. However, these mechanisms are not yet fully understood.

Empirical evidence has characterized some general properties of transitive relations. For example, response latencies increase and accuracy decreases in responses to transitive relations compared with responses to baseline relations (Bentall et al., 1993; Fields et al., 1995; Spencer and Chase, 1996). This pattern of responses is well captured under the hypothesis of different *relatedness* between the members of a class (Fields, 2016). This hypothesis argues that each pair of stimuli in a class is characterized by a particular relational strength; some pairs are more closely related than others, and these degrees of relation underlie the observed differences in response latencies, accuracy and preference for comparison stimuli. Further, studies on *nodal distance effects* show that relatedness varies as a function of the number of nodes (i.e., distance) between the members of a class. A node is defined as any stimulus that is related to at least two other stimuli during training (Fields and Verhave, 1987). For example, there could be many training structures to teach a 5-member stimulus class ABCDE. *Cluster training* (also called one-to-many training (e.g., Arntzen and Nikolaisen, 2011) consists in teaching of AB, AC, AD, and AE, whereas *linear series training* consists in teaching of AB, BC, CD, and DE. Nodal distance effects are reported when, after linear series training, relatedness appears as an inverse function of nodal distance. For example, stimulus A should be more closely related to C than to D, and so on (Fields et al., 1990, 1995; Kennedy, 1991; Spencer and Chase, 1996; Bentall et al., 1999; Bortoloti and de Rose, 2009; Moss-Lourenco and Fields, 2011; Bortoloti et al., 2013).

To date, there is no consensus about the mechanisms underlying the ability to form equivalence classes, particularly, about how transitive relations are derived, and there are no quantitative approaches to analyze and describe differences in relatedness between the members of an equivalence class. Our objective in this paper is to put forward a mechanistic explanation for the establishment of transitive relations and equivalence classes through an abstract neurocomputational model that incorporates general principles of associative learning. Before we present our model, we will briefly review the main theoretical perspectives and computational models of SE. We will also discuss why they have been insufficient in explaining the establishment of transitive relations.

### Theoretical perspectives on stimulus equivalence

Three main theories have been proposed to account for equivalence class formation. Sidman (1994, 2000) suggested that equivalence relations are a direct outcome of reinforcement. On this view, all positive elements that take part in a reinforcement contingency can become part of an equivalence class. Sidman sees equivalence as a primitive function from which other behaviors, such as naming, derive.

In contrast to the reinforcement approach, Lowe and colleagues (Dugdale and Lowe, 1990; Horne and Lowe, 1996) proposed that *naming* is what enables participants to respond correctly to SE tasks. Under this view, equivalence responding is mediated by naming skills either because stimuli in a class become equivalent by acquiring the same name, or because, having different names, they can be linked through a verbal description. Evidence for this theory comes from failed attempts to observe derived relations in nonhuman animals (Sidman et al., 1982; D'Amato et al., 1985; Campos et al., 2011).

A third perspective is based on the Relational Frame Theory (RFT) by Hayes and colleagues (Hayes, 1991; Hayes et al., 2001). This view argues that derived equivalence is a generalized form of relational responding, and thus appeals to prior experience with learning stimulus relations. Learning different stimuli as interchangeable (e.g., bidirectional relations of the kind if A then B, and if B then A) is then generalized to new stimulus sets like those presented during SE experiments.

These theories are not completely differentiable through experimental tasks. For example, in a study relating language and SE, Devany et al. (1986) analyzed the performance on transitivity probes in three groups of children. The first group consisted of four typically developing children, the second of four children with learning disability with some language skills, and the third of four children with learning disability who lacked any language skill. The results showed that all children with language skills (groups 1 and 2) responded correctly to transitive relations, whereas children without language skills did not. Even though these results seem to support the naming theory, they leave open the question if it was the absence of language that caused poor performance on SE or whether problems with language and SE had a common underlying cause. In another study, Luciano and colleagues (Luciano et al., 2007) observed equivalence responding in a typically developing 19-month-old toddler with a small verbal repertoire. The toddler participated in a *multiple exemplar training* study, consisting of learning bidirectional relations (e.g., object 1 to sound 1, and sound 1 to object 1) for a variety of objects before being trained and tested on equivalences. Her success in equivalence responding suggests that prior exposure to bidirectional relations led to equivalence formation, and therefore seems to support RFT. Nevertheless, the absence of control conditions weakens this conclusion, due to the fact that equivalence could emerge without the multiple exemplar training, fitting also into Sidman's reinforcement theory.

### Computational approaches

Substantial contributions to our knowledge about categorization have come from computational modeling. However, most computational models of categorization have focused on categories that are based, at least in part, on perceptual similarities (Kruschke, 1992; Love et al., 2004), and therefore they are not suitable for modeling the processing of equivalence classes. On the other hand, abstract formal models of learning that adequately describe relations between perceptually different stimuli have focused on the correspondence between only two stimuli (or events) as a result of stimulus co-occurrence, cue competition, and prediction, mainly in the context of Pavlovian conditioning (Rescorla and Wagner, 1972; Ramscar et al., 2010).

Only a small number of computational models have addressed some specific aspects of the learning of equivalence classes. Barnes and Hampson (1993) presented a model that aimed to assess whether derived equivalence relations could be demonstrated in connectionist networks. Their model was designed to perform matching to sample tasks in a feed forward neural network with back propagation learning. The results from their simulations seemed to demonstrate the establishment of trained and derived equivalence relations. Nonetheless, after further analyses it was argued (Tovar and Torres-Chávez, 2012) that the way the stimuli were coded in the simulations of Barnes and Hampson hindered a full demonstration of transitive responding since these relations were partially trained, thus not derived, in the neural network.

Tovar and Torres-Chávez (2012) presented a second connectionist network that demonstrated learning of trained and transitive relations. This network was designed to learn mappings between inputs representing stimulus pairings (e.g., A1B1, A1B2) and outputs representing yes/no responses. This model served to make explicit how in experimental procedures that use stimulus pairings and two response options, previous learning of input-output mappings from all the possible relations of one equivalence class (i.e., class XYZ) was required to correctly derive transitive relations of novel equivalence classes (e.g., class A1B1C1) that were only partially trained.

The reviewed connectionist networks (Barnes and Hampson, 1993; Tovar and Torres-Chávez, 2012) were limited by their inability to describe relatedness between the elements of a stimulus class. They can tell little about how relatedness increases, how it characterizes trained and derived relations, or whether and how relatedness and class formation are affected by different training procedures. The components and functioning of these models were not linked to biologically viable modules or processes; further, back propagation has been criticized as being an implausible learning mechanism in biological neurons as there is no clear evidence that an error signal can be back propagated in the brain to modify activation flowing in subsequent forward propagation (O'Reilly and Munakata, 2000).

Lew and Zanutto (2011) developed a neurocomputational model proposing some biological mechanisms that allow the establishment of trained and derived equivalence relations. In this model prefrontal and motor areas interact with visual areas through top-down processing that modulate short term memory of stimuli, then Hebbian learning and lateral inhibition are responsible for synaptic changes that consolidate learning of stimulus relations. An important drawback of this model is that it is not directly compared with empirical data. It also does not provide precise descriptions or predictions about relatedness between elements of an equivalence class; therefore, whether and how these biological mechanisms account for empirical observations of trained and transitive relations remains unknown.

Since the described theories and models appear to address only partially the SE phenomena, or without recourse to empirical data, an integrated account is still missing. Our objective in this paper is to provide such an integrated account for the formation and processing of equivalence classes through a neurocomputational model. This model seeks to incorporate equivalence phenomena into traditional accounts of associative learning; particularly, the model is based on Hebbian learning principles, and it uses the concept of spreading activation to account for emergent transitive relations and the processing of this type of categories in which exemplars have no perceptual overlap. We discuss that the learning process in our model, although abstract, is biologically motivated, and its performance is empirically valid; it is informed by current notions of synaptic plasticity and its performance is directly compared with empirical studies. In the following sections we first describe the model and then show how it accounts for a wide range of empirical results in research on SE.

## Neurocomputational model

Our neurocomputational model relies on the following simple assumptions: (1) Stimuli are represented in a localist fashion through the activation of units in a neural network. (2) Weighted connections exist between the representations for different stimuli and allow spreading activation through the network. (3) Based on Hebbian learning principles the coactivation of units results in long term changes in the connection weights. In some cases, coactivation occurs because two stimuli are present in the environment, for example, when stimulus relations are directly experienced. In other cases, coactivation results from spreading activation in the neural network through the learned connections. We hypothesize that this latter mechanism can account for relatedness of derived transitive relations.

### Architecture

**Figure 2:**
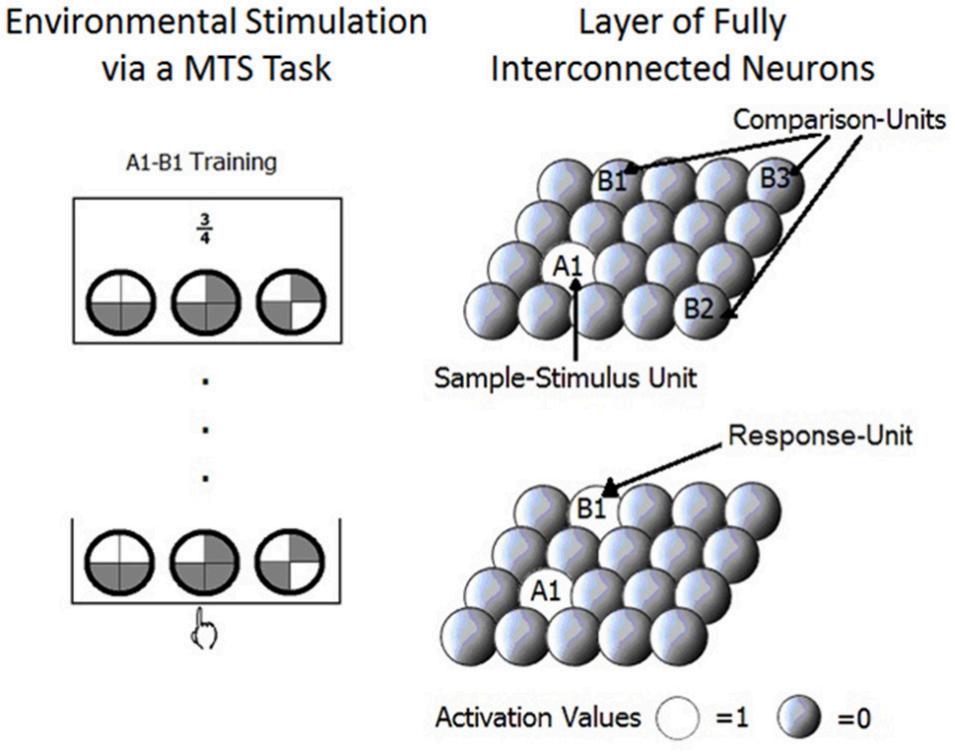
The layer of fully interconnected neurons that compose the model is showed. The sample stimulus unit, comparison units and the response unit are presented on the right side of the figure in correspondence to what happens in a matching to sample trial used with human participants and represented on the left side of the figure.

The model consists of one layer of fully interconnected neurons or units (see Figure 2). Each unit (X_1_, X_2_ …,X_n_) represents one stimulus. Given that stimuli in SE studies are chosen to minimize perceptual overlap, in the model distributed representations (that are useful for representing perceptual overlap) are not necessary. Activation values of the units range from 0 to 1.

Activation flows through connections that link the units (W_ij_,…,W_mn_). The connection weights are interpreted as the level of relatedness, or associative strength, between the stimuli represented in the corresponding units. Connections between units are bidirectional. All connection values in the model start at 0 to simulate no previous experience. Through training the connections are able to acquire any value between −1 and 1. We will now explain how this model is used to simulate learning in MTS tasks.

### Activation of units

Units are activated either through exposure to an external stimulus or through activation flowing through the connections from other units (i.e., spreading activation).

Stimuli are presented to the network as binary patterns. When a sample stimulus is presented, for example stimulus A1, the unit that represents A1 in the network is set to an activation value of 1; we will refer to this unit as the sample stimulus unit. Then comparison stimuli are presented, for example B1, B2, and B3, and the units that represent them compute the input that they receive through the connections from the sample stimulus unit (e.g., A1). The most active of these comparison units transforms its activation value to 1, becoming the response unit. The comparison stimulus represented by this unit is considered as the one selected to match the sample stimulus; the other comparison units are set to 0. This procedure simulates competition through lateral inhibition between comparison units.

Activation then propagates to the layer of units through the connections. All remaining units perform a weighted sum of all the inputs they receive from the already active units and transform this net-input into an activation/output value through a sigmoid function (see Equation 1). The activation of units is then followed by connection weight updates, and these processes form one iteration of the model.

(1) $$\begin{array}{r} \text{For net\_input}>0.85,{\text{X}}_{\text{j}}=1/1+\text{exp}{-}^{\text{net\_inputj}}\\ \text{Else }{\text{X}}_{\text{j}}=0 \end{array}$$

### Learning

Learning is captured in our model through the modification of connection weights. Once the response unit becomes active the model receives a *feedback* or *reinforcement* signal that indicates the direction for connection weight changes. We used a supervised version of Hebbian learning: in our model connection strengthening occurs after correct responses (coactivation of sample and correct comparison units), and connection weakening occurs after incorrect responses. The formalization of Hebbian learning is commonly expressed as

(2) $$\begin{array}{r} {\text{W}}_{\text{ij}}\;\left(\text{t}+1\right)={\text{W}}_{\text{ij}}\left(\text{t}\right)+\beta \left({\text{X}}_{\text{i}}^{*}{\text{X}}_{\text{j}}\right) \end{array}$$

where W_ij_ (t+1) is the value that the connection between neurons i and j acquires after adding to its previous value W_ij_(t), the result of coactivation between i and j (${\text{X}}_{\text{i}}^{*}$X_j_) weighted by β. In our model β is a learning rate with positive sign for correct responses and negative sign for wrong responses.

Note that if Equation (2) is implemented as a continuous process, the result is an endless strengthening of W_ij_, because any coactivation strengthens the connection weight which leads to more activation in a reiterative loop. To control this overgrowth we specify that strengthening of connections takes place only for a range θ…1 of coactivation values. The lower limit of this range (θ) is a coactivation threshold that is biologically motivated (see below). Coactivation values below this limit lead to decays in connection weights. We also include a self-adapting value (λ) to modulate the amount and direction of weight changes. For over-threshold coactivation values, λ is computed by subtracting Wij from coactivation. For below-threshold coactivation values, lambda acquires a negative value proportional to the current W_ij_ (see Equation 3).

(3) $$\begin{array}{r} \text{For }\left(\text{X}{\text{i}}^{*}Xj\right)>\theta ,\lambda =\left(X{\text{i}}^{*}Xj\right)-{\text{W}}_{\text{ij}}\\ \text{Else }\lambda =-{\text{W}}_{\text{ij}} \end{array}$$

Through this process we ensure that the extent and direction of changes in Wij depend on whether there is a big or small, positive or negative difference between the relatedness already learned W_ij_(t), and the current coactivation of units (${\text{X}}_{\text{i}}^{*}$X_j_). This allows us to implement a form of metaplasticity (see (Abraham, 2008) in the model, which entails modifying the current properties of weight strengthening in response to its previous history.

In the model there are two possible sources for the weakening of connections: incorrect responses, and negative values in λ. As mentioned previously a negative β is used for weight updates after incorrect responses, and a value of −β^*^0.25 is used for weight updates after negative values in λ. Equation (4) shows the Hebbian learning algorithm that includes λ and β.

(4) $$\begin{array}{r} {\text{W}}_{\text{ij}}\left(\text{t}+1\right)={\text{W}}_{\text{ij}}\left(\text{t}\right)+\left[\lambda \beta \left(X_{\text{i}}^{*}X_{\text{j}}\geq \theta \right)\right] \end{array}$$

During the simulation of testing phases the model does not receive any feedback signal. Nevertheless, minimal changes in the connection weights still occur; this was modeled by reducing β during tests to one fourth of its value during training.

### Biological plausibility of the learning algorithm

The way in which our model computes weight changes is consistent with evidence of synaptic adjustments in biological neural networks. There is strong support from studies on long-term potentiation (LTP) demonstrating that synapses display Hebbian adjustments (Bliss et al., 2007). In these studies, synaptic efficacy is observable to depend on the firing dynamics of the connected neurons. LTP describes synaptic strengthening that appears after a period of high frequency activations in pre- and post-synaptic neurons. This is captured in our model by positive changes in connection weights. When the frequency of activations in pre- and post-synaptic neurons is lower than a threshold, a reduction of synaptic efficacy is observed and described as long-term depression (LTD) (Malenka and Bear, 2004; Lüscher and Malenka, 2012) which is captured by our model by negative changes in connection weights. In our model the described lower limit (θ) that must be surpassed in order for strengthening to take place ensures that increases in the connections occur only after high coactivation values, as described by LTP after high frequency stimulation.

The self-adapting λ in our model leads to stabilizing the value of connections by switching between big or small, positive or negative changes in connection weights. This is in accordance with neurophysiological evidence of activity dependent mechanisms that regulate synaptic changes maintaining the stability of neuronal connections. These mechanisms are usually studied as part of homeostatic plasticity (Turrigiano and Nelson, 2000) and metaplasticity processes (Bliss et al., 2007; Abraham, 2008) and include, among others, regulation of synapse number, changes in neuronal excitability, and activity-dependent LTP/LTD-threshold changes.

## Modeling stimulus equivalence

We ran four simulations. These where analyzed by observing the connection values (weight matrix) between all the units representing the stimulus class members. In the equivalence literature, the performance of participants is usually analyzed through completion of criteria during training (e.g., 90% of correct trials in a block) and speed and accuracy during equivalence tests. We followed this approach for our simulations, and provided training to the model until it matched the same criteria as in the original studies. For evaluating the performance of the model during test, the connection values were interpreted as relatedness or associative strength between stimuli. Based on the empirical evidence reviewed above that shows positive correlations between relatedness and response accuracy and speed, relatedness in our model was compared to accuracy and response speed reported from the human experiments. The first simulation did not model any actual empirical study. Instead, it was designed to observe adjustments of connection values during a regular training procedure that establishes a 3-member stimulus class. It also served to fix values for parameters β and θ.

For simulations 2–4 we modeled the studies of Sidman and Tailby (1982), Devany et al. (1986), and Spencer and Chase (1996), respectively, for the following reasons: first, these studies provide clear descriptions about the procedures used for training and tests. Second, the results they show can be directly compared to the connection values in the model. And third, these studies have had a high impact on the SE literature.

We had three specific objectives. The first was to evaluate the viability of the model to account for trained and derived stimulus relations. The second was to simulate the performance of participants with disabilities to replicate failures in transitive responding, and thus to offer an explanation for both successful and failed transitivity, addressed in Simulation 3. The third objective, addressed in Simulation 4, was to evaluate if the model accounts for nodal distance effects, and hence to provide a mechanistic approach for processes than rely on the establishment of transitive relations.

### Simulation 1: AB, BC training

This simulation was designed to observe the adjustment of connection values during a simple training of two stimulus relations, and to determine the values for β and θ. Two stimulus relations were directly trained in the model: A1B1 and B1C1. Training of A1B1 began with the presentation of sample stimulus A1 along with the comparison stimuli B1, B2, and B3. After the activation of the response unit the model received a feedback signal and weights were updated. Then the next trial was presented with sample stimulus B1 along with C1, C2, and C3 as comparisons. Training continued until 30 epochs were completed. Epochs consisted of presenting each trained trial once, such that the network was trained for a total of 60 trials.

#### Results

**Figure 3:**
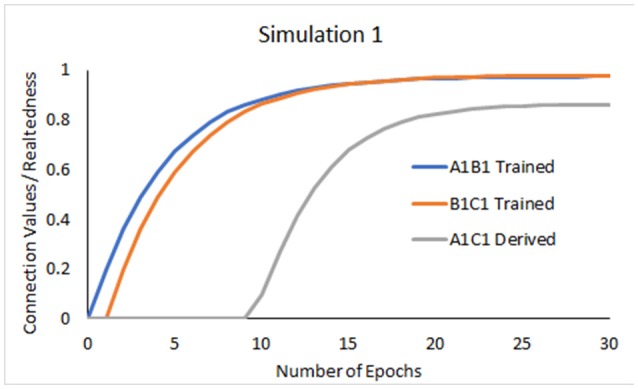
Development of connection values interpreted as the relatedness between the trained A1B1, B1C1, and derived A1C1 relations.

The model showed learning of trained relations through increases in the connection values between the units A1 and B1, and B1 and C1 (Figure 3). At some point in the training (near epoch 11), repetitions of A1B1 led the network to evoke either stimulus when the other was presented, so that e.g., A1 now also became active when B1C1 was trained. This resulted in activation of A1, B1, and C1 whenever B1 was presented and thus, in an increase of the connection strength between the coactive A1 and C1 units despite these two stimuli never being presented together.

As shown in Figure 3, relatedness between transitive A1C1 remained lower than relatedness between trained A1B1 and B1C1. Based on the assumption that relatedness manifests in accuracy and response speed, this result converges with empirical evidence showing higher accuracy, speed and preference for trained relations vs. derived transitivity relations (Bentall et al., 1993; Spencer and Chase, 1996; Moss-Lourenco and Fields, 2011).

In this simulation we set the learning parameters as follows: coactivation threshold (θ) = 0.7, and learning rate (β) = 0.2. These values were used for the remaining simulations.

### Simulation 2: Sidman and Tailby (1982)

Initial studies on SE analyzed stimulus classes containing only three members. A major purpose of Sidman and Tailby's (1982) study was to analyze the inclusion of a fourth member in a stimulus class to test the power of equivalence relations to generate a larger network of interchangeable stimuli. Participants were eight typically developing children. Stimuli were a set A of spoken Greek letter names; sets B, C, and D were sets of different printed Greek letters.

All children were trained on a total of three 4-member stimulus classes. For each class, children first went through auditory-visual MTS tasks to learn AB and AC relations. They were reinforced for choosing the printed letters from sets B or C that corresponded to the heard letter names from set A. Following this stage, they proceeded to visual-visual MTS to master the DC relations.

Training comprised several stages. The nine trained relations (AB, AC, DC, in classes 1, 2, and 3, respectively) were progressively introduced as summarized in Table 1. For example, training of AB relations was divided in four stages: (1) A1B1 and A2B2 trials; (2) A1B1 and A3B3 trials; (3) A2B2 and A3B3 trials; and (4) A1B1, A2B2, and A3B3 trials. A minimum of correct trials achieved by the children allowed them to proceed to the next stages, otherwise the stage was repeated (specific criteria for all stages are depicted in Table 1).

**Table 1:** Sequence of training and testing phases used in Sidman and Tailby (1982) and in Simulation 2.

**Sidman and Tailby, 1982**	**Simulation 2**	**Criterion**
**Training**	**Training**	**Correct/Total trials**
**1. Training of AB**	**1. Training of AB**	
A1B1, A2B2	A1B1, A2B2	19/20
A1B1, A3B3	A1B1, A3B3	19/20
A2B2, A3B3	A2B2, A3B3	19/20
A1B1, A2B2, A3B3	A1B1, A2B2, A3B3	29/30
**2. Training of AC**	**2. Training of AC**	
A1C1, A2C2	A1C1, A2C2	19/20
A1C1, A3C3	A1C1, A3C3	19/20
A2C2, A3C3	A2C2, A3C3	19/20
A1C1, A2C2, A3C3	A1C1, A2C2, A3C3	29/30
**3. Training of AB and AC**	**3. Training of AB and AC**	
A1B1, A2B2, A3B3, A1C1, A2C2, A3C3	A1B1, A2B2, A3B3, A1C1, A2C2, A3C3	29/30
**4. Training of DC**	**4. Training of DC**	
D1C1, D2C2	D1C1, D2C2	19/20
D1C1, D3C3	D1C1, D3C3	19/20
D2C2, D3C3	D2C2, D3C3	19/20
D1C1, D2C2, D3C3	D1C1, D2C2, D3C3	29/30
**5. Training of AB, AC, and DC**	**5. Training of AB, AC, and DC**	
A1B1, A2B2, A3B3, A1C1, A2C2, A3C3, D1C1, D2C2, D3C3	A1B1, A2B2, A3B3, A1C1, A2C2, A3C3, D1C1, D2C2, D3C3	44/45
**6. Gradual lowering of reinforcement probability**		
Tests^*^	Tests	Total trials in Sidman and Tailby (1982)
1. DB mixed with baseline AB, AC, and DC	One block of trials containing all baseline relations and DB, BD, AD, BC, CB, and CD test relations	120
2. BD mixed with baseline AB, AC, and DC		120
3. AD mixed with baseline AC, and DC		90
4. BC mixed with baseline AB, and AC		60
5. CB mixed with baseline AB, and AC		60
6. CD mixed with baseline DC		60
7. Naming probes		

*The third column shows the criterion established to move on the training stages for both the original study and the simulation*.

* *The stages 1–7 of Phase 6 were presented in different sequences. Only Participants A.D. and D.W. received tests in the sequence from 1 to 7*.

Once AB, BC, and DC were mastered, reinforcement probability was gradually lowered and then MTS tests for derived DB, BD, AD, BC, CB, and CD relations were presented in blocks of trials which contained also the corresponding prerequisite baseline relations; for example, since derived AD required the establishment of AC and DC, the block of trials corresponding with assessing AD contained AD tests mixed with AC and DC baseline trials. Naming probes were performed at the end; however, these were not considered for Simulation 1. During training, MTS tasks included one sample and two or three comparison stimuli; during tests all trials included one sample and three comparisons.

**Figure 4:**
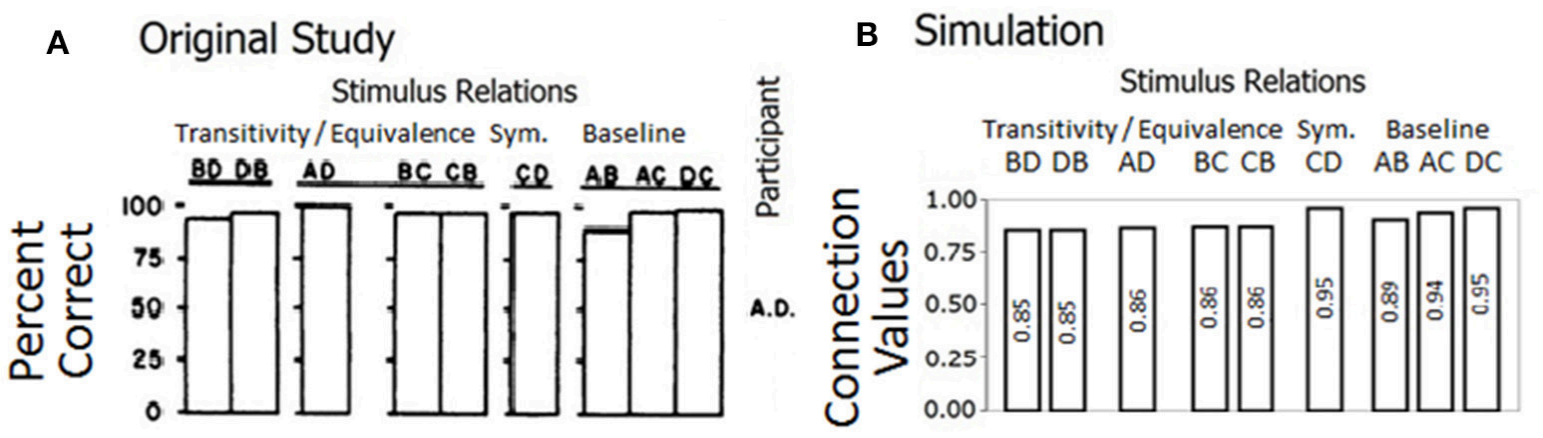
**(A)** Is reproduced from Sidman and Tailby (1982) with permission from John Wiley & Sons, Inc. Each row of bars gives participant's A.D. percentage of correct responses on transitivity/equivalence, symmetry (sym.), and baseline trials. **(B)** Shows the final connection values/relatedness in the network for derived and baseline relations.

Six out of eight children demonstrated equivalence relations between class members by responding with high accuracy levels in the test phase. Sidman and Tailby (1982) argued that the conditional-discrimination procedure used to train stimulus relations of the kind *if A then B*, generated equivalence relations. Figure 4A is taken from Sidman and Tailby (1982) and shows the percentage of correct responses for trained and tested relations for participant A.D; the other six participants who formed equivalence classes showed similar results.

To match the training of our model with the training from the original study we designed input patterns that corresponded to the nine trained relations (AB, AC, and AD for classes 1, 2, and 3). Each of these input patterns was presented in accordance with the same sequence and number of repetitions as in the original study. For example, to simulate the first stage of AB training, a block of 20 trials was presented to the model with 10 A1B1 trials mixed with 10 A2B2 trials. If the response unit activated by the model corresponded with the correct comparison stimulus in at least 19 trials, as was the criterion in the original study, then we moved on the next stage with a block of 20 trials containing 10 A1B1 mixed with 10 A3B3 trials, and so on. Table 1 shows the sequence and number of trials presented to the model.

We skipped the gradual lowering of reinforcement in the simulation; this procedure is used with human participants to maintain their performance during tests even when they do not receive any feedback. In the model, there is no need to motivate response unit activations in extinction conditions. The model only captures the impact of reinforcement on associative learning, but not on motivating performance.

To assess derived relations, we presented one block of trials with the nine trained relations and the 18 tested relations without reinforcement (Tested relations: DB, BD, AD, BC, CB, and CD for classes 1, 2, and 3). Since there is only minimal weight adaptation in the network during tests, there are no significant differences for presenting one vs. many test blocks. In the original study the authors used different sequences for presenting stimulus relations across participants during tests. We simulated the sequence used for Participant A.D. and the results of our simulation are directly compared with the results from this participant.

#### Results

The model strengthened connection values corresponding with trained and derived stimulus relations as depicted in Figure 4B. The connection values, achieved by the model, are compared with the percent of correct responses shown by Participant A.D. In the model, all stimulus relations show values above 0.85. The trained relations (AB, AC, and AD) are stronger than the transitive relations (BD, DB, AD, BC, and CB). There are slight increases from AB to AC to DC in both the connection values of the model and the percent of correct responses by Participant A.D. In the model, these differences result from the simulated sequence (see Table 1).

By replicating the results from the original study, our model captures the establishment of 4-member stimulus classes. This simulation shows the linkage between connection values in our model and accuracy levels from human participants, thus provides evidence in favor of our mechanistic approach to equivalence class formation.

### Simulation 3: Devany et al. (1986)

Devany and colleagues evaluated 12 children divided in three groups: (1) typically developing children, (2) children with a learning disability with some language skills, and (3) children with a learning disability without language skills. The children learned AB and AC relations from two classes, and then BC and CB were assessed during tests (the nomenclature was changed from the original study). Training and tests were conducted with MTS trials with two response options. Stimuli were animal-like figures. Reinforcement for correct responses included praise, access to soap bubbles and balloons.

Training was divided into 7 stages. Stimulus relations were gradually introduced in blocks of 10 trials per stage. After 9 out of 10 consecutive correct responses children moved on the next stage. Then, children received blocks of mixed trials where reinforcement probability was gradually lowered.

During tests the B1C1, C1B1, B2C2, and C2B2 relations were assessed in a block of 40 trials. Reinforcement contingent with equivalence responding was not used during tests. The stimulus relations in each training stage and tests are depicted in Table 2.

**Table 2:** Sequence of training and testing phases used in Devany et al. (1986) and in Simulation 3.

**Devany et al., 1986**	**Simulation 3**	**Criterion**
**Training**	**Training**	**Correct/Total trials**
A1B1	A1B1	9/10
A2B2	A2B2	9/10
A1B1, A2B2	A1B1, A2B2	9/10
A1C1	A1C1	9/10
A2C2	A2C2	9/10
A1C1, and A2C2	A1C1, A2C2	9/10
A1B1, A2B2, A1C1, A2C2	A1B1, A2B2, A1C1, A2C2	9 consecutive correct/10 in original study 7/8 in Simulation 3 with each relation appearing twice
		
A1B1, A2B2, A1C1, and A2C2 with gradual lowering of reinforcement probability until ≈0.25	A1B1, A2B2, A1C1, and A2C2 without reinforcement	Not specified in original study. 2/2 for each relation in Simulation 3
**Tests**	**Tests**	–
One block with B1C1, C1B1, B2C2, C2B2 tests, each relation was presented 10 times	One block with B1C1, C1B1, B2C2, C2B2 tests, each relation was presented once	

*The third column shows the criterion established to move on the training stages for both the original study*.

One highlight result in this study was that children from group 3 (learning disabilities without language skills) did not show establishment of transitive relations. They also took longer to complete the training phase. Meanwhile, the children from groups 1 and 2 acquired the trained relations in fewer training blocks and responded correctly to transitive relations.

Our computational simulation aimed to account for the performance of children in group 3. We assumed that the more or less developed language abilities, as well as the capability to learn equivalences, are both outcomes of a system with particular constraints. Therefore, we aimed to elucidate a possible mechanism in the context of Hebbian learning that accounted for deficits in deriving stimulus relations.

Neurophysiological evidence from mouse models of intellectual disability (e.g., models of Down syndrome and Fragile X syndrome) has shown altered plasticity processes, particularly increased levels of synaptic weakening (LTD) and reduced levels of strengthening (LTP) (Rueda et al., 2012). It has been suggested that an impaired balance between LTD and LTP may result from an increased threshold to produce LTP, so that neural mechanisms associated with LTP induction are in place but require higher levels of activity to be triggered (Meredith et al., 2007). A number of synaptic alterations that impact on computing power have been also described for populations with intellectual disability. The main alterations include reduction of synapse density, inhibitory predominance, and abnormal growth of dendritic spines (Dierssen et al., 2003; Ayberk Kurt et al., 2004; Dierssen, 2012).

The evidence about an altered LTP/LTP balance motivated a modification in our model to simulate participants in group 3 by increasing the coactivation threshold θ, which is functionally analogous to the LTP threshold in that it restricts the connection strengthening in the model to high coactivation values, and we lowered the learning rate β, which captures more generally the synaptic abnormalities that limit computing power. We ran the model once with the regular parameters to simulate typically developing children from group 1. In a second step, to simulate children with learning disability from group 3, we increased the coactivation threshold θ from 0.7 to 0.72; and lowered the learning rate β from 0.2 to 0.1. Children from group 2 were not considered in this simulation since they have a profile of learning disability that seems less altered than the profile of children from group 3.

For the simulation we presented input patterns corresponding to the 4 trained relations with the same sequence and the same number of trials used in the original study (see Table 2). During tests, four input patterns representing the four tested relations (i.e., B1C1, C1B1, B2C2, and C2B2) where presented to the model in a randomized sequence.

#### Results

**Figure 5:**
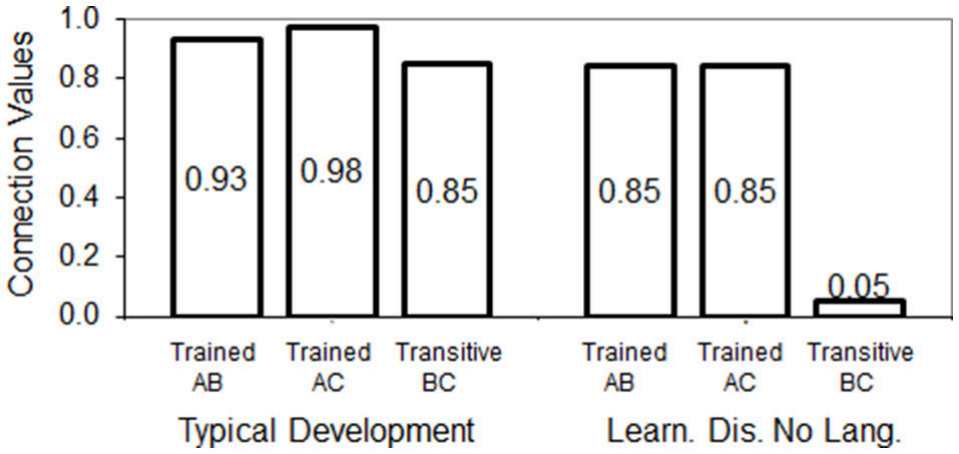
Connection values/relatedness achieved by the model for trained and tested relations when typical development and learning disability (Learn. Dis. No Lang.) groups are simulated.

The main result from Devany et al. (1986) was that typically developing children from group 1 responded with high accuracy to transitive relations approaching an errorless performance as the test trials went on, while children from group 3 (learning disabilities without language skills) performed near chance level during tests. We calculated the mean accuracy during tests from the graphics presented in the original paper for groups 1 and 3. Group 1 showed 85% of correct responses to transitive relations, while group 3 performed near chance level with 42% of correct responses to transitive relations. In Figure 5 we show in bars the connection values achieved for trained and derived relations when the conditions of typical development and learning disability were simulated.

Both models show learning of the baseline relations AB and AC, although the connection values are stronger in the simulation of typically developing children compared with the learning disability simulation. The connection value for transitive relations in the simulation of typical development (0.85) approaches the mean accuracy of the typically developing children in group 1 (85.6%). In contrast, in the learning disability simulation, transitive weights failed to develop almost completely. This accounts for the chance-level performance of children from group 3.

The mechanistic explanation of failed transitivity in the model is as follows: direct stimulation (training trials) generated full activation of units and resulting coactivation was likely to surpass the threshold to trigger weight strengthening. However, connection weights did not develop as strong as in the simulation of typical development, thus for transitive relations, the coactivation values resulting from spreading activation were less likely to surpass the elevated threshold and trigger positive changes in the corresponding weights. This is, once AB and AC were trained, the connections of A with both B and C allowed the network to activate units A, B, and C for any given training trial, but it was unable to strengthen the transitive connection between B and C since they produced a coactivation value that fell below the threshold.

#### Further simulations and model prediction

Further simulations in our model of learning disability indicated that establishing transitive relations was possible by using an alternative training sequence. A possible cause for weak connections of baseline relations is that the training of one relation (AB) interferes with training of the second (AC). During AC training the network not only strengthens the connection between A and C but also weakens (decays) the connection between A and B, due to the fact that AB coactivation is lower than θ. Nonetheless, in this model, when AB is trained alone (avoiding interference of AC) until its connection acquires a value near 1 (i.e., 0.99 after 45 trials) and just then blocks of mixed AB and AC trials are presented (30 trials), the presentation of an AC trial generates activations of A, B, and C with values that now surpass the threshold and the transitive BC relation appears in the network.

These simulations can be taken as a prediction of our model indicating that avoidance of interference during training should benefit trained and derived equivalence relations in participants with learning disabilities.

### Simulation 4: Spencer and Chase (1996)

Different relatedness between the members of a stimulus class may depend on nodal distance: as the number of nodal stimuli increases the relatedness decreases. Decreased relatedness is supposed to manifest in increased response times. In their study, Spencer and Chase were particularly interested in measuring the response speed during equivalence responding. Their purpose was to characterize baseline and derived relations through temporal analyses of responding.

In the original study there were three experimental groups. The first group was verbally instructed about how stimuli were related and the second was queried during testing about the rationale of their responses. For this simulation we focus on the third group called *standard group*, formed by college students who were neither instructed nor queried about their responding. Stimuli were 21 nonsense figures arranged in three 7-member stimulus classes. Participants learned six sets of relations (AB, BC, CD, DE, EF, and FG for classes 1, 2, and 3) via MTS with three response options per trial. Training was divided into seven stages with 48 trials per stage. Each set of relations was learned during each of the first six stages; for example, the AB set, composed of A1B1, A2B2, and A3B3 relations, was learned during the first stage, and once completed, participants moved on to the second stage. From the second to the sixth stage 24 trials were used for new stimulus relations and 24 were designated for maintenance of previously learned relations. Correct responses were followed by verbal feedback and gaining of points; each point was equivalent to $0.01. Incorrect responses resulted in the darkening of the screen for one second and no gain of points. The accuracy criterion to advance from one stage to the next was at least 90% of correct trials with no more than one error on any one relation. During stage 7 all the learned relations were intermixed in the 48-trial block and presented in extinction conditions. Five consecutive blocks of stage 7 were required to be responded to with at least 90% of correct trials to finish training. Table 3 shows the stimulus relations and sequence used for the 7 training stages.

**Table 3:** Sequence of training and testing phases used in Spencer and Chase (1996) and in Simulation 4.

**Spencer and Chase**, **1996**							**Simulation 4**							**Criterion**
**Training stages**	**Number of trials per relation**						**Training stages**	**Number of trials per relation**						
	**AB**	**BC**	**CD**	**DE**	**EF**	**FG**		**AB**	**BC**	**CD**	**DE**	**EF**	**FG**	
1. AB	48						1. AB	48						90% correct
2. BC	24	24					2. BC	24	24					90% correct
3. CD	12	12	24				3. CD	12	12	24				90% correct
4. DE	8	8	8	24			4. DE	8	8	8	24			90% correct
5. EF	6	6	6	6	24		5. EF	6	6	6	6	24		90% correct
6. FG	3	3	3	6	9	24	6. FG	3	3	3	6	9	24	90% correct
7. Baseline maintenance. No reinf.	3	3	3	3	3	3	7. Baseline maintenance. No reinf.	3	3	3	3	3	3	90% correct in 5 consec. blocks in original study. 90% correct in one block in Simulation 4
**Test Stages**							**Test Stage**							
1. Combined	36 baseline trials						Combined, Transitivity and Symmetry	18 baseline trials						90% correct in original study. No criterion in Simulation 4
	45 combined trials							18 symmetry trials						
2. Transitivity	36 baseline trials							45 combined trials						
	45 transitivity trials							45 transitivity trials						
	15 combined trials													
3. Symmetry	36 baseline trials													
	18 symmetry trials													
	15 combined trials													
	15 transitivity trials													

*The third column shows the criterion established to move on the training stages for both the original study and the simulation*.

A total of 108 derived relations were evaluated: forty-five transitive relations (e.g., A1C1), another 45 equivalence trials called “combined trials” because symmetry and transitivity were combined (e.g., C1A1) and 18 symmetry trials (i.e., B1A1). These trials are listed in Table 4. Three stages of testing evaluated a different proportion of test trials. The number and type of trials evaluated during these stages are depicted in Table 3. No reinforcement was presented during tests, however, at least 90% of correct trials were required in each test stage to continue and finish the experiment. Spencer and Chase (1996) found a tendency to decreased response speed as nodal distance increased, demonstrating nodal distance effects.

**Table 4:** All the stimulus relations presented as baseline, symmetry, transitivity or combined trials in Spencer and Chase (1996) and in Simulation 4.

**Type of Trial**	**Stimulus relations presented for classes 1, 2, and 3**
Baseline	AB, BC, CD, DE, EF, FG
Symmetry	BA, CB, DC, ED, FE, GF
Transitivity	AC, AD, AE, AF, AG, BD, BE, BF, BG, CE, CF, CG, DF, DG, EG
Combined	CA, DA, EA, FA, GA, DB, EB, FB, GB, EC, FC, GC, FD, GD, GE

Training in the network was based on the number of trials, sequence of stages and criteria from the original study. A total of 18 input patterns were designed to teach the six sets of relations (AB, BC, CD, DE, EF, and FG) for the three classes. Stage 7 presented all the trials randomly mixed without reinforcement. During tests the network was stimulated with a total of 126 input patterns which corresponded to the total of possible trials (Table 4); 18 baseline, 18 symmetry, 45 transitive and 45 combined trials.

#### Results

**Figure 6:**
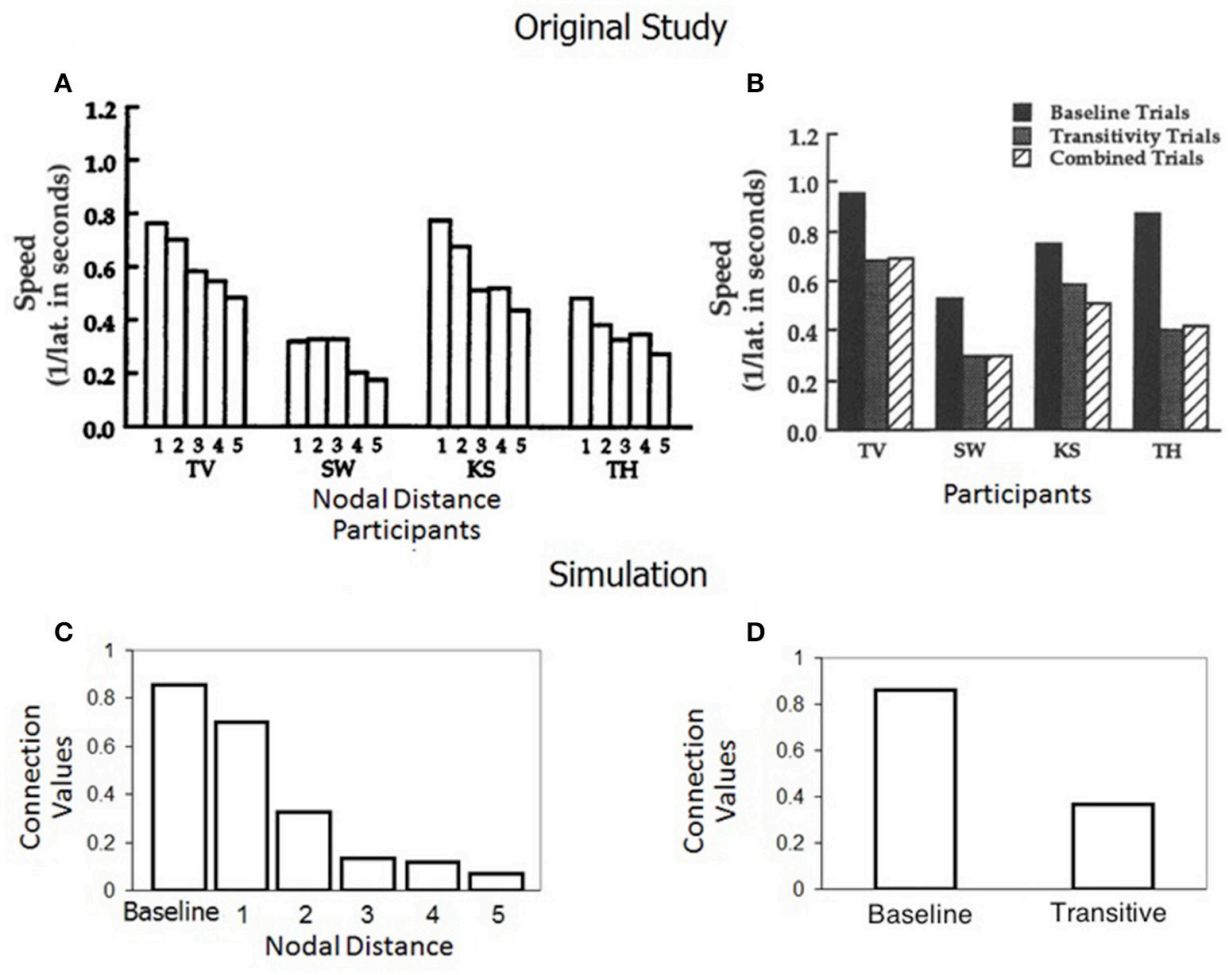
**(A,B)** Are taken from the standard group of participants in Spencer and Chase (1996) with permission from John Wiley & Sons, Inc. **(A)** Shows the mean speed of correct responding on one through five node transitivity trials. **(B)** Shows the mean speed of correct responses on baseline, transitivity and combined (symmetry plus transitivity, or equivalence tests) trials. **(C)** Shows the final mean connection values/relatedness in Simulation 4 for baseline and one through five node transitivity relations. **(D)** Shows the final mean connection strength values/relatedness for baseline and for all transitive relations in Simulation 4.

Figures 6A,B show results from the original paper, particularly the response speed for baseline and derived relations with a tendency toward decreasing speed as nodal distance increases. Figure 6C shows the values of the connection weights of the model for baseline and derived relations depending on the number of nodes separating each stimulus relation. It can be observed that relatedness appears as an inverse function of nodal distance. As the number of nodes increases, the level of association between stimuli in a class decreases. Figure 6D shows the mean connection values achieved by the model for all baseline and all derived relations.

#### Further simulations exploring transitive relatedness

Although the relatedness between baseline and one through five-node transitivity relations reported by our model was in line with the speed analyses reported by Spencer and Chase, we were interested in testing if there were other influences, besides nodal distance, that could explain the final relatedness between stimuli. This question was in part motivated by the idea that nodal distance can be confounded with exposure to a different number of baseline trials (Imam, 2006).

The left side of Table 3 shows the number of trials scheduled for each baseline relation when the original study of Spencer and Chase was simulated. Since baseline relations were gradually introduced and mixed with maintenance trials of previously learned relations, there was a considerable difference in the frequency of presentation for each trained relation. For example, AB relations were presented more frequently than the rest of the trained relations.

We ran a simulation in which we trained the model with 65 trials of each of the AB, BC, CD, DE, EF, and FG relations. The relatedness for baseline and one through five node transitive relations achieved by the model is presented in Table 5 and values are compared with the relatedness obtained when the training phase of Spencer and Chase was simulated. Differences in relatedness still appear in accordance with nodal distance, however, there is not a gradual decrease in relatedness; instead one and two node relations achieved the same value of 0.77 which is just below the relatedness value of baseline relations (0.78), three node relations acquired a value of 0.68, and there is no derived transitivity for four and five node relations.

**Table 5:** Relatedness for baseline and one through five node transitive relations reported by the neurocomputational model after simulating the training procedure by Spencer and Chase (1996) and simulating training with equal numbers of trials per trained relation.

**Simulation of Spencer and Chase (****1996****) procedure**		**Simulation with equal number of trials per trained relation**	
**Stimulus relation**	**Relatedness**	**Stimulus relation**	**Relatedness**
Baseline	0.83	Baseline	0.78
1-node	0.7	1-node	0.77
2-nodes	0.33	2-nodes	0.77
3-nodes	0.13	3-nodes	0.68
4-nodes	0.12	4-nodes	0
5-nodes	0.07	5-nodes	0

Our model therefore shows that nodal distance may not be the only source of differential relatedness for transitive relations in a stimulus class, this also partially explains why there is a non-continuous degradation of associative strength as nodal distance increases, suggesting that relatedness also depend on other variables such as different frequency of trained relations.

## Discussion

We have presented a neural network model that simulated several core results from the SE literature, focusing on exploring relatedness for trained and transitive relations. The learning mechanism incorporated in our model, based on Hebbian learning, extracted the statistics of co-occurring events in the environment by means of adapting artificial synaptic connections between coactive stimulus representations, which accounted for relatedness of trained relations. Our hypothesis is that the model accounts for how human participants are sensitive to the environmental regularities of stimulus correlations, which may have a strong influence on how they learn, and organize information of stimulus classes.

The Hebbian learning mechanism in our model is biologically sound. The inclusion of parameters θ and λ, which modulate the positive and negative changes of connection efficacy, were motivated by neurophysiological descriptions of functionally analogous processes, including LTP/LTD balance and metaplasticity (Bliss et al., 2007; Meredith et al., 2007; Abraham, 2008). Notably, the way in which these parameters operate, in combination with our instantiation of a reinforcement signal through the learning rate β, is in accordance with traditional approaches of reinforcement learning. For example, in the Rescorla and Wagner model (Rescorla and Wagner, 1972) the associative strength between two stimuli changes depending on the difference between the prediction of co-occurrence and the actual co-occurrence of stimuli. In our model, changes in connection weights result from the co-occurrence of sample and comparison stimuli after correct and incorrect responses. Thus, for trained stimulus relations, the quantitative description of associative strength provided by our model is similar to that suggested by the Rescorla and Wagner model. Our model, however, extends the scope and explanatory power of reinforcement learning models by providing quantitative descriptions of associative strength for derived (non-directly experienced) stimulus relations.

Deriving the transitive AC relation after being trained on AB and BC was explained through spreading activation triggering the Hebbian learning mechanism: whenever B was presented, activation flowed through non-zero connections and triggered activation of the representations of A and C. Therefore, with A, B, and C coactive as an outcome of a mixture of direct stimulation and spreading activation, relatedness between A and C was learned. Our model, then, presents a parsimonious approach to equivalence since it accounts for trained and transitive relations through the same principles. Moreover, it integrates the study of equivalence classes with a wider approach of associative and reinforcement learning. In support of this view, we showed that transitive relatedness in our simulations is comparable with accuracy and response speed in three published empirical studies, covering processing in typical and atypical children, and nodal distance effects.

Our model did not require the learning of previous abilities or experience with other sets of stimuli to show learned and derived relations, which are prerequisites in the naming and RFT approaches to SE (Horne and Lowe, 1996; Hayes et al., 2001). Sidman's (1990) account considers equivalence as a basic stimulus function not derivable from more fundamental processes. At this point our model converges with Sidman's account in that equivalence is a basic process, and we propose a mechanistic explanation for trained and derived transitive equivalences based on Hebbian learning principles and spreading activation processes. Nonetheless, we cannot exclude that experience and development of other symbolic behavior can have an influence on equivalence formation.

The simulation of Sidman and Tailby (1982) covered the performance of typically developing children showing final connection weights that were in accordance with accuracy performance of the children. The simulation of Devany et al. (1986) showed that the model is also suitable to simulate performance of children with atypical development. This was done by adapting the neural coactivation threshold in accordance with neurophysiological hypotheses of increased LTP thresholds in models of learning disability (Meredith et al., 2007). When disabilities were simulated the model learned the trained relations but could not derive transitive relations as the children from the original study did. A prediction of the model, and a possibility for intervention in children with learning disabilities, arose by using a training procedure that was slightly different from the one of Devany and colleagues. This showed that if stimulus relations are mastered avoiding interference, by means of exhaustive training of one stimulus relation before its training is mixed with another stimulus relation, then transitivity appears.

Simulation 4 replicated nodal distance effects after linear series training when the procedure by Spencer and Chase (1996) was simulated. The model showed that relatedness for transitive relations depend on the number of nodes and number of trials used for training.

Compared with previous computational models of equivalence classes (Barnes and Hampson, 1993; Lew and Zanutto, 2011; Tovar and Torres-Chávez, 2012), our approach provides a quantitative description for trained and transitive relations that can be directly compared with speed and accuracy of participants in empirical studies. As a key contribution, our model provided a tool to explore the effects of different training procedures on learning and deriving stimulus relations. One implication of this approach is that it allows further testing by confirming our predictions for trained and derived equivalence relations after specific training schedules. The second implication is that with this model it will now be possible to run simulations to test the influence of trial repetitions, sequence of trained relations, training structures, and other variations in order to find the best way to teach equivalence relations and thereby propose training protocols to be used with people, either typically developing or with learning disabilities. This will make it easier for participants to expand a number of behavioral skills related to using equivalence classes with benefits in comprehension, reading, mathematics, and general symbolic communication skills.

In our model stimulus relations are learned through a purely bottom-up process based on the co-occurrence of stimuli in the environment and the activation and modification of lateral connections between stimulus representations, and we show that these processes are sufficient to account for a range of behaviors in the processing of equivalence classes. However, top-down processes can also modulate class formation (Wisniewski and Medin, 1994). For example, if a participant experiences the stimuli AB together along with the label “not related,” it is probable that she does not derive transitive equivalence between A and other stimuli associated with B. Such “meta-information” cannot be represented in the current model. Our model also does not account for shifts in the functional properties of stimulus relations, for example as a result of changing the context, where for example, A1*R*B1 in context one, but A1*R*B2 in context two. Likewise, in the model we used localist representations in order to minimize perceptual overlap between stimuli so that SE formation was solely based on co-occurrence statistics and not on perceptual relatedness. While this choice was based on the existing literature using stimuli that are not perceptually similar, as a consequence it is impossible in the model to integrate effects of perceptual overlap. For this to become possible distributed representations would be necessary.

In this model, we provided a biologically motivated learning algorithm for the learning of equivalence classes. Aiming for parsimony we kept the architecture of our model simple (i.e., a single layer of fully interconnected units). Future models should evaluate if a more complex architecture that incorporates the learning principles presented here could significantly extend the scope of this approach. Our model, so far provides a mechanistic account of the learning and derivation of SE relations, for both typical and atypical populations with potential implications for studies on categorization, organization of semantic memory, and concept formation.

## Author contributions

AT and GW developed the computational model. AT ran the simulations. AT and GW analyzed the data and prepared the manuscript.

### Conflict of interest statement

The authors declare that the research was conducted in the absence of any commercial or financial relationships that could be construed as a potential conflict of interest.
